# Impact of Transferrin Saturation and Anemia on Radial Artery Calcification in Patients with End-Stage Kidney Disease

**DOI:** 10.3390/nu14204269

**Published:** 2022-10-13

**Authors:** Toshiki Kano, Hiroaki Io, Junichiro Nakata, Yu Sasaki, Masahiro Muto, Yuki Shimizu, Yusuke Fukao, Haruna Fukuzaki, Takuya Maeda, Reina Hosoya, Yusuke Suzuki

**Affiliations:** 1 Department of Nephrology, Juntendo University Nerima Hospital, Tokyo 177-8521, Japan; 2 Department of Nephrology, Juntendo University Faculty of Medicine, Tokyo 113-8421, Japan

**Keywords:** radial artery calcification, end-stage kidney disease, arteriovenous fistula

## Abstract

Background: Arterial calcification is an important factor in determining the prognosis of patients with chronic kidney disease (CKD). Few studies on aortic calcification have involved radial artery calcification (RAC). This study aimed to analyze risk factors for RAC in patients with end-stage kidney disease (ESKD) and investigate the relationship between subsequent cardiovascular events (CVE) and vascular access trouble (VAT). Methods: This cohort study included 64 consecutive patients with ESKD who initiated hemodialysis and underwent a procedure for the creation of a primary radiocephalic arteriovenous fistula (RCAVF). Small arterial specimens were obtained from patients during RCAVF surgery. Tissue samples were stained with von Kossa, and arterial microcalcification was evaluated. We analyzed the association between preexisting arterial microcalcifications, clinical characteristics, CVE, and VAT. Results: In the univariate analysis, RAC patients demonstrated high systolic blood pressure (sBP), low hemoglobin (Hb), and low transferrin saturation (TSAT) (<0.05, <0.05, and <0.05, respectively). In the multivariate analysis, Hb (HR–0.516 (0.278–0.959), *p* < 0.05), TSAT (HR–0.0012 (0.00000248–0.597), *p* < 0.05), and sBP (HR–1.037 (1.001–1.073), *p* < 0.05) were independent risk factors for RAC. The cumulative incidence rate of CVE/VAT was not associated with RAC for one year. Conclusion: RAC was associated with sBP, TSAT, and anemia; however, no association with CVE/VAT was observed.

## 1. Introduction

Vascular calcification is broadly divided into two types: intimal and medial calcification [1]. Intimal calcification is often found in atherosclerosis, which is associated with diabetes and dyslipidemia. Medial calcification is characteristic of patients with chronic kidney disease (CKD) [2]. Clinically, the presence and extent of vascular calcifications have been reported as strong predictors of cardiovascular and all-cause mortality [3,4].

Previous studies have shown that hyperphosphatemia is closely associated with medial vascular calcification in patients undergoing hemodialysis [5]. Dietary phosphate overload directly induces chronic inflammation, malnutrition, and vascular calcification in renal failure model rats [6]. A hyperphosphatemic environment affects the cellular fate of vascular smooth muscle cells (VSMCs) by delivering phosphate into VSMCs through phosphate transporters, such as phosphate transporter 1 (Pit-1), Pit-2, and calciprotein particles [7]. Phenotypic differentiation into osteogenic/chondroblast-like VSMCs occurs following diverse signaling pathways that sensitize VSMCs to calcification [8]. The uremic rat model induced vascular calcification with increased Runt-related transcription factor 2 (RUNX2) in the vascular tissue by phosphorus loading. Therefore, phosphorus loading is considered a direct factor in cell transformation [9]. In addition, it has been shown that oxidative stress, which increases in a toxic urinary state, induces cell transformation through the induction of cellular apoptosis [10,11].

To date, there have been several studies evaluating arterial calcification using diagnostic imaging, such as computed tomography (CT), in patients with end-stage kidney disease (ESKD) or hemodialysis patients; however, few studies have pathologically evaluated arterial calcification. Patients with ESKD have a lot of complications, such as anemia due to iron deficiency or the impaired use of iron. A previous report showed that oxidative stress contributes to hypoproliferative renal anemia by causing the peroxidation of membrane lipids and impairing erythropoietin action [12]. It is useful to investigate whether complications such as anemia further promote arterial calcification via phosphorus. In addition, studies have not investigated the cause of radial artery calcification and the subsequent incidence of cardiovascular events (CVE). Understanding the risk factors for this calcification and the subsequent clinical course may improve the prognosis of patients with ESKD through prevention and treatment. Thus, in this study, vascular access trouble (VAT) and CVE after an arteriovenous fistula (AVF) operation were investigated based on the presence or absence of radial artery calcification (RAC) using a part of the arterial tissue that was collected when the native AVF was prepared in ESKD patients. A stepwise analysis was also performed on the biochemical laboratory findings that caused RAC.

## 2. Materials and Methods

### 2.1. Study Overview

We designed a cohort study of 64 consecutive patients with ESKD who initiated hemodialysis and underwent a primary radiocephalic arteriovenous fistula (RCAVF) procedure in the forearm between July 2015 and March 2019 at Juntendo University Hospital and Juntendo University Nerima Hospital, Tokyo, Japan, and they were followed up for 12 months. A small arterial specimen was obtained during AVF surgery from patients who provided informed consent. The obtained tissue samples were stained with von Kossa and evaluated for arterial microcalcification by two pathologists blinded to the patients’ clinical information. Finally, we evaluated the association between preexisting arterial microcalcifications, clinical characteristics, and clinical CVE/AVF outcomes.

### 2.2. Procedures

Baseline data on age, sex, the presence of diabetes mellitus (DM), and past cardiovascular disease (CVD) were obtained from the institutional database. Blood pressure (BP), hemoglobin (Hb), transferrin saturation (TSAT), ferritin, albumin, creatinine, estimated glomerular filtration rate (eGFR), high-sensitivity C-reactive protein (hsCRP), intact parathyroid hormone, alkaline phosphatase, albumin-adjusted serum calcium (Ca), phosphate, total cholesterol, low-density lipoprotein (LDL) cholesterol, high-density lipoprotein (HDL) cholesterol, triglyceride concentrations, oral iron use, phosphate binder use, erythropoiesis-stimulating agent (ESA) use, renin–angiotensin system inhibitor (RAS-I) use, and the duration of ESKD (the duration of ESKD was defined as the period from the first indication of eGFR < 15 mL/min/1.73 m^2^ to the initiation of hemodialysis) of each participant were measured immediately before their RCAVF operation. Biochemical analyses were performed at the hospital laboratory. All of the procedures performed in this study were in accordance with the ethical standards of institutions and national research committees and the 1964 Declaration of Helsinki with subsequent amendments or equivalent ethical standards. The protocol was approved by the Ethics Committee of Juntendo University Hospital and Juntendo University Nerima Hospital, Tokyo, Japan (No. 14-196), and registered with the University Hospital Medical Information Network (UMIN 000019385). Informed consent was obtained from all participants. Based on Japanese guidelines, we ensured that iron administration was adequate in all participating patients [13].

### 2.3. Pathological Studies of Arterial Specimens

**Figure 1:**
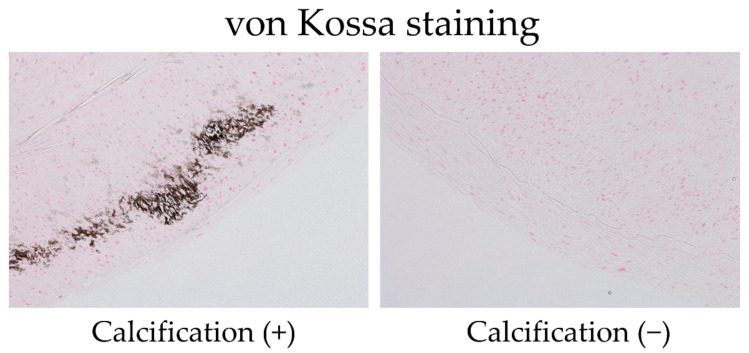
Representative von Kossa stains of arterial specimens obtained during arteriovenous fistula (AVF) surgery. Calcium stains are black. The pathologists evaluated arterial specimens with or without calcification. The left panel displays an artery with calcification, and the right panel illustrates an artery with no calcification.

All arterial specimens obtained during AVF surgery were immediately fixed in 10% neutral buffered formalin. They were sectioned as 50% planes from paraffin-embedded blocks using a TRUSTOME (Japan) microtome. The specimens were stained using the von Kossa (Japan) method for calcium quantification, which can detect microcalcifications. The slides were placed in a 5% silver nitrate solution in water, and the calcium cations were replaced with silver, turning the mineralized material black. After being placed in 5% sodium thiosulfate, the specimens were counterstained using Kernechtrot (Germany) for contrast, which selectively stains nuclear chromatin red and provides nonspecific background tissue staining in shades of pink (Figure 1).

In short, von Kossa staining reveals calcium deposits. Tissue sections are treated using a silver nitrate solution, which is deposited by replacing calcium and then reduced by strong light, thereby visualized as metallic silver.

Arterial microcalcifications were assessed by two pathologists who consented to this study and were blinded to the patient’s clinical characteristics and AVF outcome. The microcalcifications were localized almost exclusively in the media, with few intima or adventitia.

### 2.4. Surgical Technique

The surgical operation was performed under local anesthesia with 1% lidocaine containing 0.001% epinephrine. A light-angle incision was made, and the cephalic vein (CV) and radial artery (RA) were isolated as distally as possible from the forearm. The CV was dilated using a heparinized saline injection. The ends of the CV were ligated, and their lateral walls were anastomosed to the lateral wall of the RA using 6-0 polypropylene in a smooth loop configuration of 6 mm diameter. Suturing was initiated using a posterior suture, followed by an anterior suture.

### 2.5. Follow-Up

Each patient was followed up for 12 months. We contacted the satellite dialysis clinic where patients who had undergone an operation between July 2015 and March 2020 underwent hemodialysis therapy via email. We obtained information on AVF failures and CVD events, including the date of the first intervention to maintain or restore blood flow and mortality.

The interval between the time of AVF surgery and the first intervention to maintain or restore blood flow was defined as the patency time.

### 2.6. Statistical Analysis

The patency rate was estimated using the Kaplan–Meier technique. The log-rank test examined differences in patency rates between the two groups. Univariate analyses of categorical variables were performed using a Chi-square test, and multivariate analyses were performed using the Cox proportional hazards model. Data are expressed as mean ± standard deviation (M ± SD). Probability values of <0.05 were considered significant. All statistical analyses were performed using the Windows version of the JMP 13.2.1 software (SAS Institute Inc., Cary, NC, USA).

## 3. Results

### 3.1. Patient Characteristics

We investigated differences in the characteristics and laboratory findings of ESKD patients with and without RAC. The clinical characteristics of all patients and the two groups that were defined according to the presence or absence of RAC (RAC+, *n* = 29; RAC−, *n* = 35) are shown in Table 1. The primary renal diseases were diabetic nephropathy (*n* = 29, 44.6%), nephrosclerosis (*n* = 12, 18.6%), chronic glomerulonephritis (*n* = 8, 12.5%), polycystic kidney disease (*n* = 3, 4.7%), other diseases (*n* = 3, 4.7%), and unknown conditions (*n* = 9, 14.0%). The average age at AVF operation was 66.8 ± 14.1 years, and 70.3% of all patients were male (45/64). The disease etiology of 42.6% of all patients was DM, and 41.5% of all patients had a history of CVD events. The average BP measured in the supine position prior to the operation was 136.6 ± 17.8/66.7 ± 17.8 mmHg.

As shown in Table 1, patients with RAC had lower TSAT (*p* < 0.05), lower hemoglobin (*p* < 0.05), higher systolic blood pressure (sBP) (*p* < 0.05), and higher serum phosphorous concentrations (*p* < 0.05) than those without RAC. Age; sex; the prevalence of diabetes; diastolic blood pressure (dBP); serum concentrations of ferritin, albumin, creatinine, hsCRP, intact parathyroid hormone (int PTH), alkaline phosphatase, correct calcium, total cholesterol, LDL cholesterol, HDL cholesterol, and triglycerides; eGFR; oral iron use; phosphate binder use; ESA use; RAS-I use; and duration of ESKD did not differ significantly between the two groups. Interestingly, these results suggest that phosphorus, low TSAT, anemia, and hypertension contribute to radial artery calcification.

### 3.2. Factors Contributing to Radial Artery Calcification

We used multivariate analysis to determine which factors independently affected RAC. The results of the logistic regression analysis of all patients, which aimed to identify the factors associated with the presence of RAC, are shown in Table 2. In the univariate logistic regression analysis, the presence of RAC was significantly associated with sBP, Hb, and TSAT; however, there were no associations with other variables. Serum phosphorus levels in patients with RAC tended to be higher than those in patients without RAC; however, the difference was not significant (*p* = 0.051). In the multivariate analysis, Hb (HR 0.516 (0.278–0.959), *p* < 0.05), TSAT (HR 0.0012 (0.00000248–0.597), *p* < 0.05), and sBP (HR 1.037 (1.001–1.073), *p* < 0.05) were independent risk factors for the presence of RAC. Therefore, we concluded that low TSAT, anemia, and hypertension independently contributed to radial artery calcification in patients with ESKD.

### 3.3. Clinical CVE/AVF Outcomes after the AVF Operation

**Figure 2:**
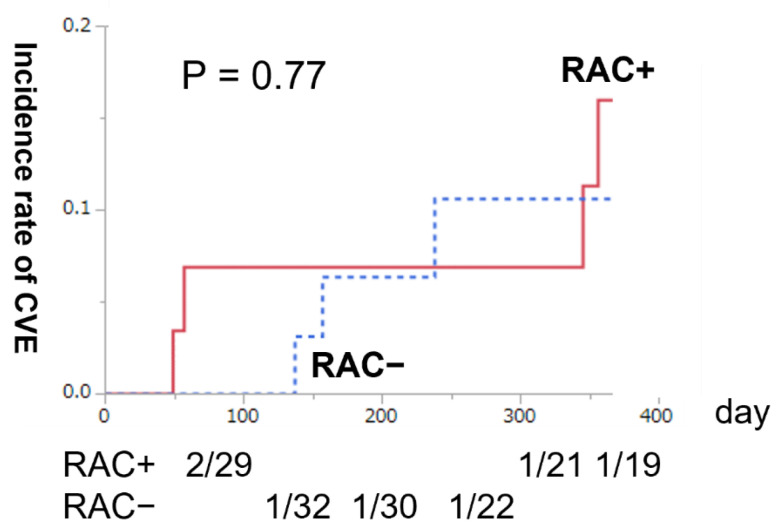
The Kaplan–Meier 1-year cumulative incidence rate of cardiovascular disease (CVD) after arteriovenous fistula (AVF) surgery. The solid red line represents radial arteries with calcification (RAC+) (*n* = 29), and the blue dotted line reveals radial arteries without calcification (RAC−) (*n* = 35). No significant difference was observed in the 1-year cumulative incidence rate of CVD between patients with and without redial artery calcification (Wilcoxon test, *p* = 0.74).

**Figure 3:**
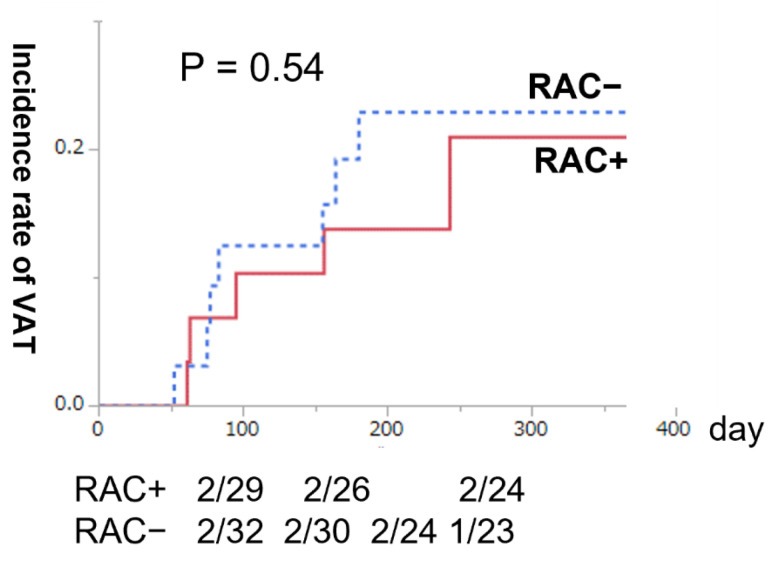
The Kaplan–Meier 1-year cumulative incidence rate of vascular access trouble (VAT) after arteriovenous fistula (AVF) creation. The solid red line presents radial arteries with calcification (RAC+) (*n* = 29), and the blue dotted line displays radial arteries without calcification (RAC−) (*n* = 35). No significant difference was observed in the 1-year cumulative incidence rate of VAT between patients with and without radial artery calcification (Wilcoxon test, *p* = 0.89).

Finally, we investigated whether the presence or absence of RAC affected the patency rate of AVF and the subsequent incidence of VAT. Among the subset of patients with mature AVF, unassisted AVF survival after maturation was similar between patients with and without RAC (Wilcoxon test, *p* = 0.74) (Figure 2). In addition, the Kaplan–Meier 1-year cumulative incidence rate of VAT was similar between patients with and without RAC (Wilcoxon test, *p* = 0.89) (Figure 3). Therefore, we observed that RAC in patients with ESKD did not contribute to the AVF patency rate or subsequent CVE over at least a year.

## 4. Discussion

It has been clarified that hyperphosphatemia is closely associated with medial vascular calcification, and it is reported that phosphorus load induces medial vascular calcification directly through PIT-1 [5,14]. Oxidative stress, which increases under uremic conditions, induces cell transformation into osteogenic/chondroblast-like VSMCs through cellular apoptosis [10,11]. In a vascular calcification model induced by phosphorus loading in uremic rats, the calcification of blood vessels and increased RUNX2 expression in vascular tissue were confirmed by phosphorus loading. This indicates that phosphorus loading directly affects cell transformation into osteogenic/chondroblast-like VSMCs [9]. In this study, serum phosphorus levels in patients with RAC tended to be higher than those in patients without RAC; however, the difference was not significant (*p* = 0.051). Since the significant difference was marginal, phosphorus levels might be significant in a larger cohort. Thus, we cannot exclude the possibility of a potential phosphorus effect or other factors that are relevant to medial vascular calcification.

In this study, we demonstrated that a decreased TSAT level was an independent risk factor for arterial calcification. A previous report showed that the intraperitoneal administration of iron suppressed vascular calcification in phosphorus-loaded uremic rats by decreasing the expression of Pit-1, RUNX2, and serum fibroblast growth factor 23 (FGF23) [9]. It was also confirmed that calcification decreased in phosphorus-loaded uremic rats treated with iron [9]. Furthermore, iron administration suppresses the induction of osteoblast gene expression by activating ferroxidase activity [15]. Serum iron levels from 60–120 ng/mL and TSAT from 30–50% were associated with the lowest all-cause and cardiovascular death risks in ESKD patients [16], and the best survival was associated with high serum iron >10.7 μmol/L, TSAT > 25%, and serum ferritin > 600 μg/L [17]. Therefore, moderate iron administration and management are important for arterial calcification and mortality in ESKD patients. On the other hand, we could not demonstrate a significant difference in ferritin levels. Ferritin is regulated by various factors, such as inflammation, malignant tumors, and blood diseases. Thus, the KDIGO guidelines suggest that TSAT is the most commonly used measure of available iron for erythropoiesis and recommend iron administration in anemic patients with CKD with TSAT < 30% (and serum ferritin < 500 ng/mL). Inflammation contributes to arterial calcification through various mechanisms, such as reducing circulating fetuin-A concentration [18]. Furthermore, inflammation is associated with high hepcidin concentrations, thereby increasing ferritin concentrations [19]. Accordingly, ferritin values have to be interpreted with caution in patients with CKD in whom subclinical inflammation may be present. Although no significant correlation was found between serum CRP and ferritin levels in this study, it was thought that subclinical inflammation, such as minor infections or tumors, may have elevated serum ferritin. In addition, CKD patients are in a state of chronic inflammation due to increased inflammatory cytokines, such as IL-6 and tumor necrosis factor (TNF)-α, in the blood as renal function declines [20].

Anemia was also an independent factor for arterial calcification, according to our results. A previous study showed that the progression of arterial calcification is associated with anemia [21]. Anemia causes peripheral hypoxia, which strongly enhances elevated inorganic-phosphate-induced VSMC calcification and osteogenic transdifferentiation, as seen through osteogenic marker expression [22]. Anemia caused by erythropoiesis-stimulating agent hyporesponsiveness is associated with a higher risk of all-cause mortality in ESKD patients as well [23,24]. Therefore, these findings suggest that anemia, arterial calcification, and mortality are associated with ESKD patients [25]. However, excessive iron intake induces reactive oxygen species formation and promotes the local formation of oxidized LDL and arteriosclerosis through the induction of macrophages; caution should be exercised in iron supplementation.

We also demonstrated that sBP is a risk factor for RAC. It is unclear whether the cause is arterial calcification or sBP because medial calcification and hypertension are intimately linked and interact. Hypertension is an important calcification-promoting factor [26]. Medial calcification decreases the elasticity of the artery, resulting in arterial stiffening, which accelerates pulse wave velocity and widens the pulse pressure, leading to hypertension [27]. In this study, there was no significant difference in dBP or mean BP, only in sBP. Arterial stiffening is principally associated with isolated systolic hypertension [28], which is consistent with our results. Previous studies have revealed only a modest association between arterial stiffness and atherosclerosis [29]. As a result, this observed association between arterial calcification, arterial stiffness, and hypertension is primarily due to medial calcification.

These findings suggest that understanding and targeting the mechanisms of medial calcification may offer new therapies. Medial vascular calcification is linked to hypertension, hyperphosphatemia, low TSAT level, and anemia, which may also account for the overlap in their clinical manifestations.

Even though we found that low Hb/TSAT and high sBP were independent risk factors for RAC in patients with ESKD, there was no difference in the patency rate or the incidence of CVE over one year. Regarding the AVF patency rate, in a previous study, vascular calcification did not affect the patency rate, which is consistent with this study [30]. However, many studies have reported that the calcification of blood vessels affects the prognosis. Previously, we reported that hemoglobin levels were associated with the left ventricular mass index beyond 12 months after HD initiation. The treatment of hypertension and anemia may slow the left ventricular mass index progression and help identify patients on hemodialysis at high risk of CVE [31]. CVE may be improved by anemia and hypertension treatments after the onset of HD. Therefore, it is necessary to examine the long-term course in the future.

This study has several limitations. First, the detailed mechanism of RAC is unknown because this was an observational study. Few published studies regarding RAC provide much evidence that RAC indicates systemic calcification. However, it is considered that arterial calcification does not occur locally but uniformly throughout the body. Second, although this study showed that RAC was not associated with CVE, the observation period was as short as one year, and long-term observation is particularly necessary. Third, we could not confirm that high phosphorus levels contributed to arterial calcification, unlike previous studies [32]. However, since the significance is marginal (*p* = 0.051), we believe that phosphorus levels could be significant in a larger cohort. Fourth, due to the small sample size of this study, further research is needed in the future.

In conclusion, we found that low Hb/TSAT and high sBP were independent risk factors for RAC in patients with ESKD. These findings lead to new insight into novel approaches to prevent calcification in patients with ESKD. We will continue to conduct observational studies over a longer period.

## Figures and Tables

**Table 1:** Comparison of baseline characteristics between patients with ESKD with and without vascular calcification.

Characteristics	Patients with RAC	Patients without RAC	*p*-Values	All Patients
(at the initiation of HD)	*n* = 29	*n* = 35		
Age (year)	64.2 ± 16.9	69.1 ± 11.2	0.17	66.8 ± 14.1
Male sex (%)	75.8	65.7	0.37	70.3
DM (%)	51.7	34.4	0.18	42.6
Past CVD event (%)	34.5	42.9	0.5	39.1
sBP (mmHg)	142.0 ± 18.4	131.8 ± 17.1	<0.05	136.6 ± 17.8
dBP (mmHg)	67.4 ± 11.4	66.1 ± 7.6	0.59	66.7 ± 9.6
Hb (g/dL)	8.8 ± 1.2	9.5 ± 1.2	<0.05	9.1 ± 1.2
Fe (μg/dL)	43.3 ± 16.7	54.5 ± 34.3	0.11	49.4 ± 28.1
TSAT (%)	20.1 ± 8.1	26.0 ± 13.0	<0.05	23.1 ± 11.0
Ferritin (ng/mL)	174.5 ± 144.1	186.9 ± 221.2	0.79	181.3 ± 190.2
Albumin (g/dL)	3.07 ± 0.53	3.16 ± 0.48	0.5	3.12 ± 0.50
Creatinine (mg/dL)	7.88 ± 2.16	7.16 ± 1.91	0.17	7.49 ± 2.05
eGFR (mL/min/1.73 m^2^)	6.25 ± 1.67	6.61 ± 2.09	0.46	6.50 ± 1.94
HsCRP (mg/dL)	0.47 ± 0.86	0.65 ± 1.10	0.52	0.57 ± 0.99
Int PTH (pg/dL)	251.8 ± 197.1	180.6 ± 141.7	0.09	212.8 ± 168
Alkaline phosphatase (U/L)	233.9 ± 78.1	330.0 ± 419.2.4	0.23	286.5 ± 314.8
Correct calcium (mg/dL)	9.06 ± 0.43	9.19 ± 0.67	0.37	9.12 ± 0.57
Phosphorous (mg/dL)	5.42 ± 1.43	4.80 ± 1.05	<0.05	5.09 ± 1.25
Total cholesterol (mg/dL)	160.6 ± 37.6	154.1 ± 35.1	0.5	157.2 ± 36.4
LDL cholesterol (mg/dL)	84.7 ± 5.14	78.8 ± 4.97	0.41	81.7 ± 27.7
HDL cholesterol (mg/dL)	42.7 ± 13.9	43.9 ± 14.4	0.73	43.3 ± 14.2
Triglyceride (mg/dL)	117.9 ± 56.3	106.3 ± 33.8	0.33	111.9 ± 46.1
Oral iron use (%)	20.7	17.1	0.64	18.6
Phosphate binder use (%)	44.8	25.7	0.11	34.4
ESA use (%)	86.2	77.1	0.68	81.3
RAS-I use (%)	72.4	71.4	0.70	71.9
Duration of ESKD (mouth)	21.6 ± 3.36	22.6 ± 2.91	0.83	22.1 ± 2.19

RAC: radial artery calcification; AVF: arteriovenous fistula; DM: diabetes mellitus; CVD: cardiovascular disease; sBP: systolic blood pressure; dBP: diastolic blood pressure; Hb: hemoglobin; TSAT: transferrin saturation; eGFR: estimated glomerular filtration rate; HsCRP: high-sensitivity C-reactive protein; Int PTH: intact parathyroid hormone; LDL: low-density lipoprotein; HDL: high-density lipoprotein; ESA: erythropoiesis-stimulating agent; RAS-I: renin–angiotensin system inhibitor.

**Table 2:** Risk factors of vascular calcification using the Cox proportional hazards model.

	Univariate		Multivariate	
Parameters	HR (95% CI)	*p*-Value	HR (95% CI)	*p*-Value
Age (per 1 year)	0.975 (0.941–1.011)	0.16		
Male versus female	0.61 (0.203–1.833)	0.37		
DM	2.05 (0.729–5.734)	0.17		
Past CVD event	0.702 (0.253–1.940)	0.49		
sBP (per 1 mmHg)	1.033 (1.0025–1.0654)	0.0328	1.037 (1.001–1.073)	0.0328
dBP (per 1 mmHg)	1.015 (0.962–1.071)	0.581		
Hb (per 1 g/dL)	0.597 (0.371–0.960)	0.023	0.516 (0.278–0.959)	0.0217
Fe (per 1 μg/dL)	0.984 (0.965–1.004)	0.11		
TSAT (per 1%)	0.0043 (0.000018–1.00687)	0.03	0.0012 (0.0000248–0.597)	0.0173
Ferritin (per 1 ng/mL)	0.999 (0.997–1.002)	0.199		
Albumin (per 1 g/dL)	0.702 (0.253–1.946)	0.494		
HsCRP (per 1 mg/dL)	0.831 (0.474–1.456)	0.508		
Int PTH (per 1 pg/dL)	1.0026 (0.999–1.0057)	0.091		
Alkaline phosphatase (per 1 U/L)	0.998 (0.996–1.0011)	0.191		
Correct Calcium (per 1 mg/dL)	0.654 (0.260–1.643)	0.358		
Phosphorous (per 1 mg/dL)	1.53 (0.972–2.417)	0.051	1.51 (0.818–2.775)	0.1638
Total cholesterol (per 1 mg/dL)	1.005 (0.991–1.019)	0.491		
LDL cholesterol (per 1 mg/dL)	1.008 (0.989–1.027)	0.401		
HDL cholesterol (per 1 mg/dL)	0.993 (0.958–1.030)	0.722		
Triglyceride (per 1 mg/dL)	1.006 (0.994–1.017)	0.324		

DM: diabetes mellitus; CVD: cardiovascular disease; sBP: systolic blood pressure; dBP: diastolic blood pressure; Hb: hemoglobin; TSAT: transferrin saturation; HsCRP: high-sensitivity C-reactive protein; Int PTH: intact parathyroid hormone; LDL: low-density lipoprotein; HDL: high-density lipoprotein.

## Data Availability

The datasets generated and/or analyzed during the current study are available from the corresponding author on reasonable request.
